# Supplementary material for: Characterization of Four Medically Important Toxins from Centruroides huichol Scorpion Venom and Its Neutralization by a Single Recombinant Antibody Fragment
Source: Toxins (Basel). 2022 May 26;14(6):369. doi: 10.3390/toxins14060369 (PMC9227038; doi:10.3390/toxins14060369)
Supplement: Supplementary file 1 [file toxins-14-00369-s001.zip › toxins-1715974-supplementary.pdf]

Supplementary Materials: Characterization of Four Medically Important Toxins from *Centruroides huichol* Scorpion Venom and Its Neutralization by a Single Recombinant Antibody Fragment

Hugo Valencia-Martínez, Timoteo Olamendi-Portugal, Rita Restano-Cassulini, Hugo Serrano-Posada, Fernando Zamudio, Lourival D. Possani, Lidia Riaño-Umbarila and Baltazar Becerril

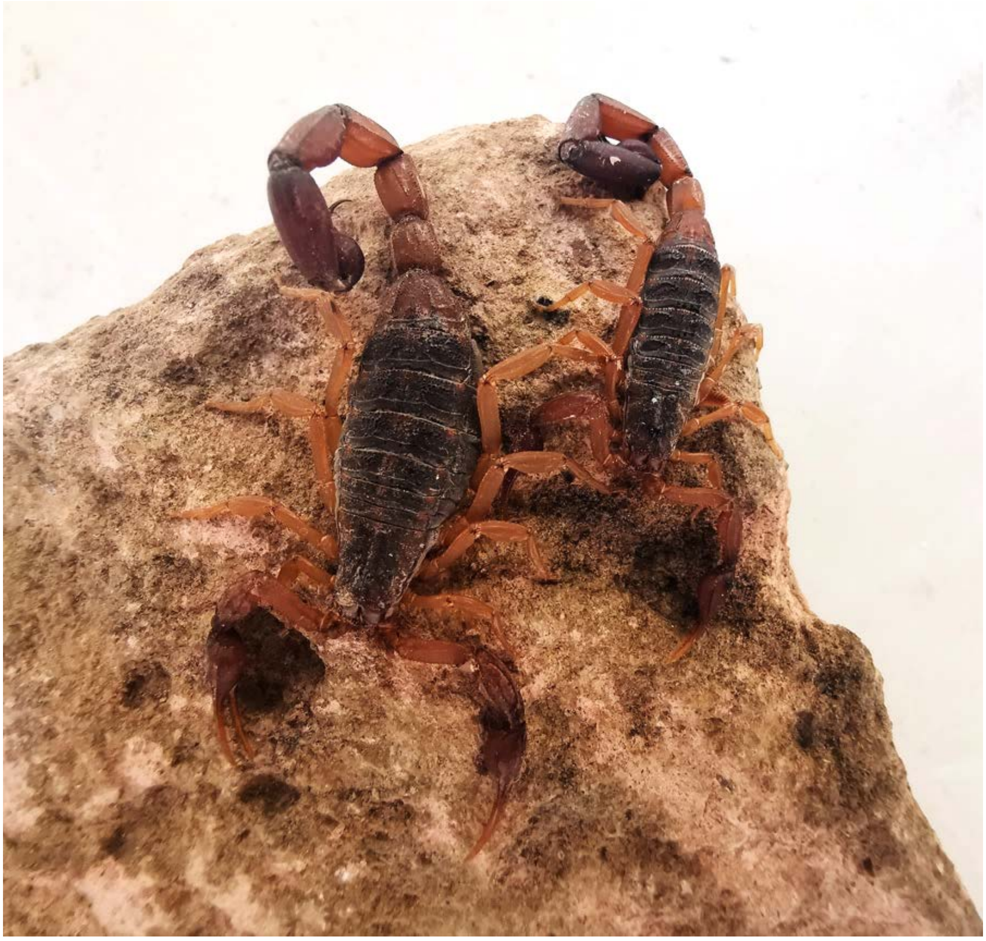

Figure S1. Representative specimens of *Centruroides huichol* scorpion. On the left side of the image, there is a female specimen and on the right side a male one. Note the sexual dimorphism represented mainly in size, with female being larger than male. Specimens collected in the municipality of San Blas, Nayarit, Mexico.

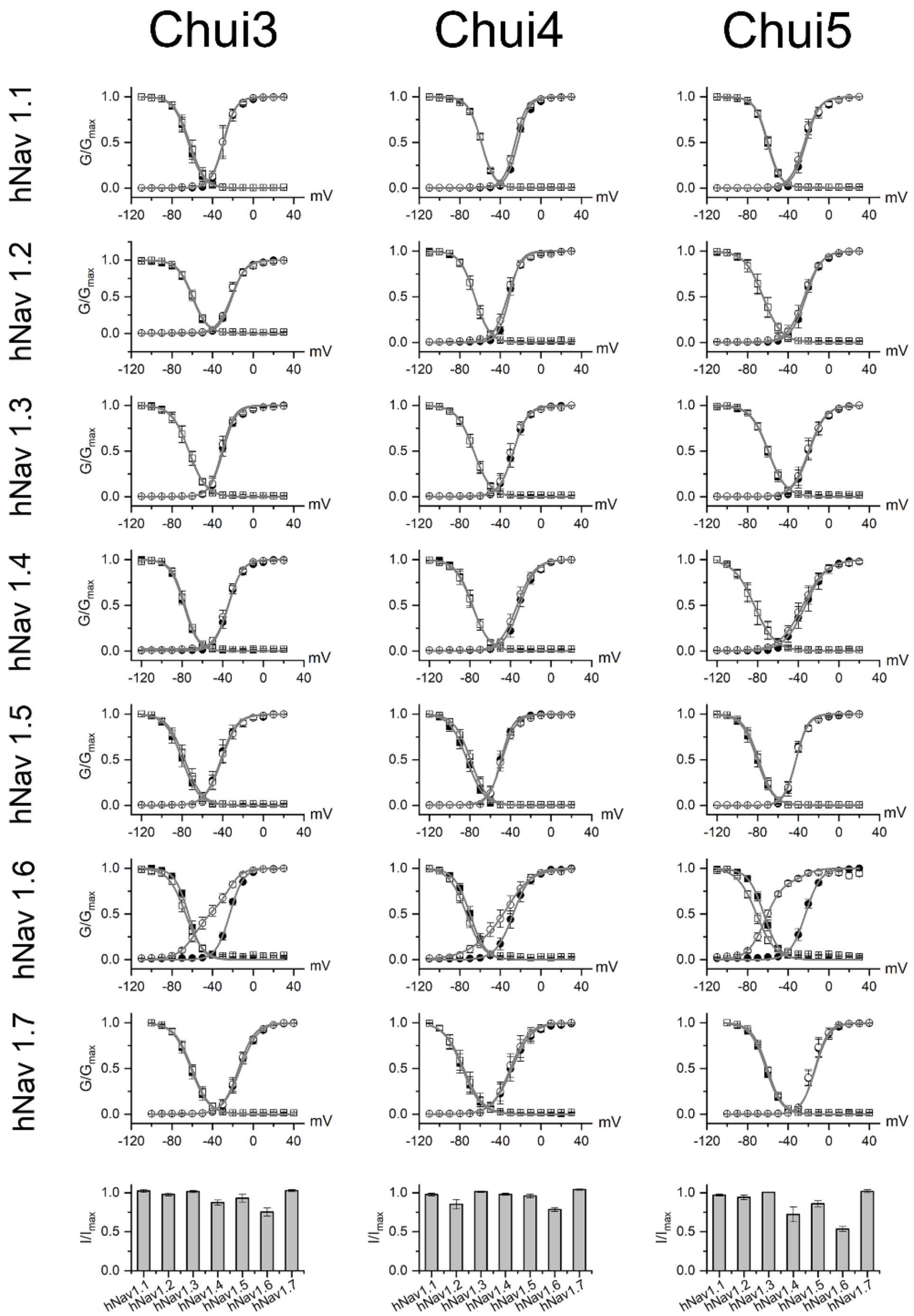

Figure S2. Effect of *C. huichol* toxins on sodium channels. The figure shows the voltage dependence of activation (circles) and inactivation (squares) process of the hNav 1.1 to 1.7 channels under control condition (closed symbols) and for toxins at 200 nM (open symbols). Data were fitted by means of Boltzmann equation (grey line). For hNav 1.6 with Chui3, Chui4, and Chui5 toxins, data were fitted with double Boltzmann equations, where one of these has the same parameter of the corresponding control condition. In the bottom row are graphed the residual current after 200 nM application of Chui3 (left panel), Chui4 (middle panel), and Chui5 (right panel). Currents were elicited during full activation potential and were normalized to the control. The activation and inactivation fitting parameters, as well as the fractional residual current values are summarized in Supplementary Table S1.

Table S1. Fitting parameters of activation and inactivation curves and residual currents after hNav1.1-1.7 channels exposition to 200 nM *C. huichol* toxins.

			hNav 1.1	hNav 1.2	hNav 1.3	hNav 1.4	hNav 1.5	hNav1.6	hNav 1.7
**Chui3**	activation	$V_{0.5}$ (mV)	−29 ± 0.4	−20.6 ± 1.8	−29.7 ± 0.5	−34.4 ± 0.3	−41.2 ± 0.5	−23.1 ± 0.3	−12.5 ± 1.9
			−29.6 ± 0.2	−21.5 ± 1.8	−30.8 ± 0.5	−35.9 ± 0.2	−41 ± 0.4	−56.7 ± 1*	−14.3 ± 1.8
		slope	6.1 ± 0.3	6.5 ± 0.6	6.5 ± 0.4	6.8 ± 0.3	7.8 ± 0.5	6.8 ± 0.2	8.6 ± 0.6
			6.1 ± 0.2	7.2 ± 0.8	6.1 ± 0.4	7.5 ± 0.2	8 ± 0.4	9.2 ± 0.6*	8 ± 0.6
		A	n.a.	n.a.	n.a.	n.a.	n.a.	0.58 ± 0.02	n.a.
	inactivation	$V_{0.5}$ (mV)	−63.8 ± 0.1	−60.1 ± 0.3	−63.4 ± 0.2	−78.2 ± 0.3	−79.9 ± 0.3	−63.8 ± 0.5	−61.3 ± 0.2
			−62.5 ± 0.1	−59.5 ± 0.2	−63.3 ± 0.3	−77.1 ± 0.3	−77.5 ± 0.3	−66.5 ± 0.7*	−60.2 ± 0.3
		slope	7.4 ± 0.1	7.4 ± 0.3	8.4 ± 0.2	6.9 ± 0.2	8.4 ± 0.3	6.9 ± 0.4	8.7 ± 0.2
			7.6 ± 0.1	7.4 ± 0.2	8.6 ± 0.2	6.8 ± 0.3	8.6 ± 0.3	8.5 ± 0.6*	8.6 ± 0.2
		I/I_{max}	1.02 ± 0.02	0.98 ± 0.02	1.01 ± 0.01	0.87 ± 0.03	0.93 ± 0.05	0.75 ± 0.05*	1.03 ± 0.01
		N	3	4	5	5	4	4	5
**Chui4**	activation	$V_{0.5}$ (mV)	−22.7 ± 0.3	−31.1 ± 0.4	−26.5 ± 1.6	−30.7 ± 0.4	−49.4 ± 0.3	−27.3 ±0.3	−28.4 ± 0.4
			−25.3 ± 0.2*	−32.9 ±0.4	−26.2 ± 1.7	−33.4 ± 0.4	−48.2 ± 0.4	−43.5 ± 2.2*	−30.1 ± 0.4
		slope	5.7 ± 0.3	5.6 ± 0.4	7.1 ± 0.4	8.1 ± 0.4	6 ± 0.3	8.6 ± 0.3	9.8 ± 0.4
			5.7 ± 0.2	6.7 ± 0.4	7.9 ± 0.5	8.6 ± 0.3	7 ± 0.3	16.5 ± 0.6*	9.3 ± 0.3
		A	n.a.	n.a.	n.a.	n.a.	n.a.	0.73 ± 0.08	n.a.
	inactivation	$V_{0.5}$ (mV)	−58.9 ± 0.2	−65.3 ± 0.2	−66.4 ± 0.3	−77.5 ± 0.3	−82.9 ± 0.3	−70.1 ± 0.4	−77.4 ± 0.4
			−58.7 ± 0.3	−65 ± 0.3	−66.3 ± 0.3	−77.8 ± 0.4	−79.4 ± 0.3	−73.4 ± 0.6*	−76.2 ± 0.4
		slope	6.4 ± 0.2	7.2 ± 0.2	8.5 ±0.2	8.2 ± 0.3	8.4 ± 0.3	7.8 ± 0.4	9.2 ± 0.3
			6.6 ±0.2	7.4 ± 0.3	8.3 ± 0.3	8.5 ± 0.3	8.1 ± 0.3	8.6 ± 0.5*	9.6 ± 0.3
		I/I_{max}	0.97 ± 0.02	0.85 ± 0.06	1.01 ± 0.01	0.98 ± 0.01	0.96 ± 0.02	0.78 ± 0.02*	1.04 ± 0.01
		N	6	6	6	5	5	6	5
**Chui5**	activation	$V_{0.5}$ (mV)	−22.5 ± 0.5	−22.4 ± 0.3	−19 ± 0.4	−25.2 ± 0.7	−42.2 ± 0.5	−23 ±0.3	−13.2 ± 2
			−23.9 ± 0.5	−24.1 ± 0.2	−20 ± 0.4	−37.3 ± 0.3	−42.2 ± 0.4	−62.6 ± 1.6*	−13.7 ± 2.4
		slope	6.7 ± 0.5	7.6 ± 0.3	8.1± 0.4	10.7 ± 0.6	5.7 ± 0.4	7.5 ± 0.3	6.9 ± 0.7
			7.2 ± 0.4	8.3 ± 0.2	8.2 ± 0.3	13.3 ± 0.3	6.1 ± 0.4	8.6 ± 1.2	6.6 ± 0.7
		A	n.a.	n.a.	n.a.	n.a.	n.a.	0.87± 0.05	n.a.
	inactivaiton	$V_{0.5}$ (mV)	−60.6 ± 01	−64.7 ± 0.2	−59.7 ± 1.6	−81.8 ± 0.3	−80.5 ± 0.2	−63.8 ± 0.6	−61.8 ± 0.2
			−59.9 ± 0.2	−64.6 ± 0.3	−59.4 ± 1.5	−84.1 ± 0.5	−79 ± 0.2	−71.1 ± 1*	−60.4 ± 0.3
		slope	6.3 ± 0.1	9 ± 0.3	8.7 ± 0.8	9.8 ± 0.2	7.7 ± 0.2	7.7 ± 0.5	7.8 ± 0.2
			6.4 ± 0.1	9.1 ± 0.2	8.6 ± 0.7	11.2 ± 0.5	7.7 ± 0.2	9.4 ± 0.9*	7.6 ± 0.2
		I/I_{max}	0.97 ± 0.01	0.94 ± 0.03	1.004±0.003	0.62 ± 0.12	0.85 ± 0.04*	0.53 ± 0.03 *	1.02 ± 0.02
		N	6	6	4	5	5	6	3

Table reports the values of the Boltzmann functions that describe steady-state activation and inactivation. Values corresponding to control condition are in no-shadow rows while values corresponding to toxin are in light grey shadow rows. $V_{0.5}$ (mV) is the membrane potential of middle activation or inactivation. In those cases when the sum of two Boltzmann functions was used to fit the steady-state activation after the toxin exposure, "A" represents the area of the function that describes the activation voltage dependence in the channels bounds to the toxin. When steady-state activation was better described by one Boltzmann function, A value was not available (n.a.). I/I_{max} is the residual current after toxin application. Data are the mean of n records ± standard error. * Indicates significant difference at 0.05 level calculated by a paired sample t assay.

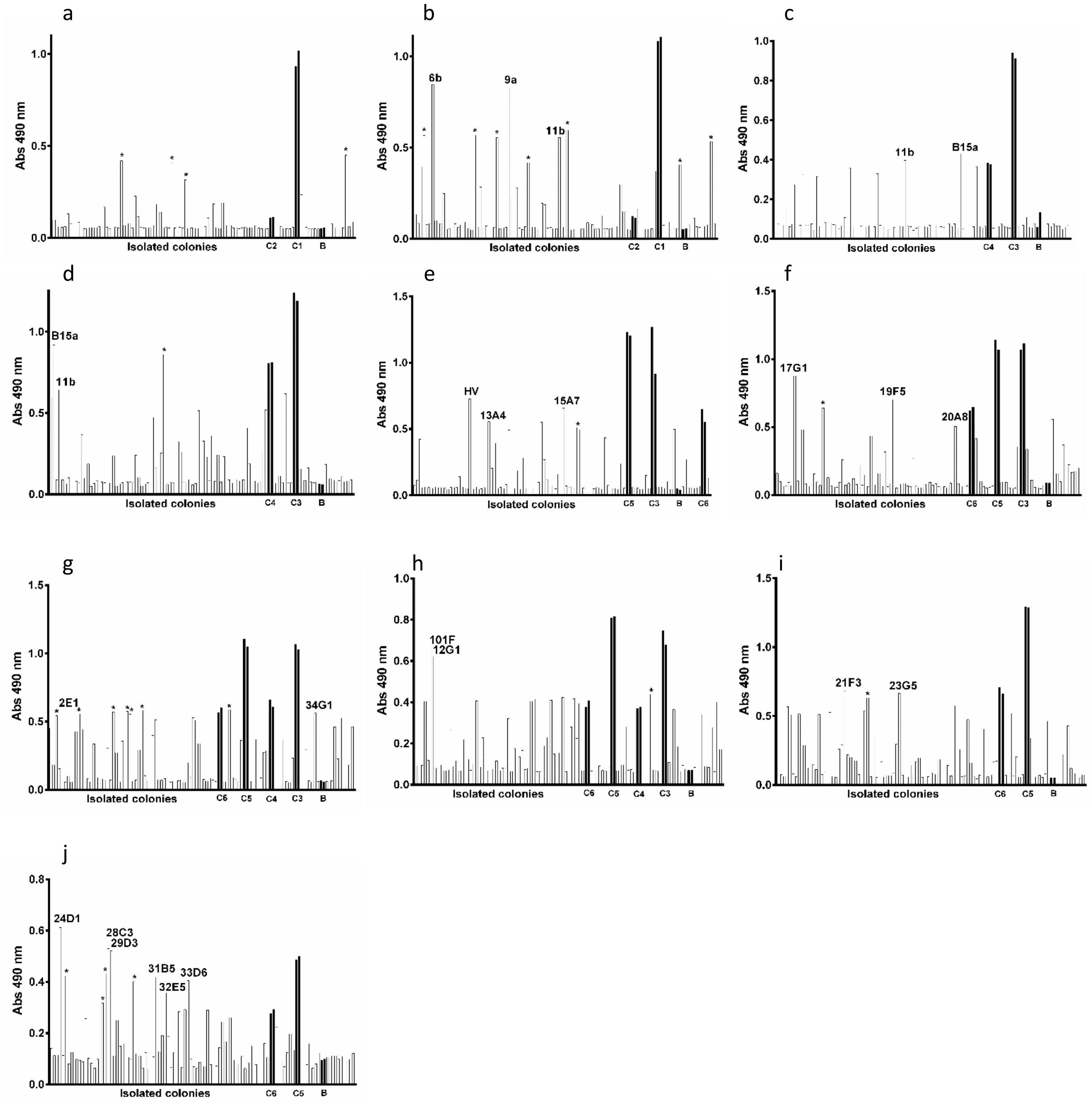

Figure S3. Evaluation by ELISA of colonies isolated from different mutagenic libraries. Soluble scFv (pSyn2; display vector) was expressed in the supernatant of randomly selected colonies from the third round of selection from: 1) 10FG2 library with site-directed mutagenesis at positions 53, 57, 59, 102, and 105 (a,b); 2) 9a library with random mutagenesis (c, d) and 3) 9a library with random and site-directed mutagenesis at positions 65 and 105 (e, f, g, h, i and j). Its interaction with Chui5 toxin was evaluated by ELISA. Colonies that were selected for further characterization are marked with an asterisk. The clones that were sequenced have their name written at the top. B: Toxin blank, background. Controls; C1: pSyn1 (expression vector) scFv 10FG2; C2: pSyn2 scFv 10FG2; C3: pSyn1 scFv 9a; C4: pSyn2 scFv 9a; C5: pSyn1 scFv B15a; C6: pSyn2 scFv B15a.

a

Process	10FG2	HFR1 1 Q	HFR1 10 G	HFR1 11 V	HCDR1 29 F	HFR2 39 Q	HCDR2 58 K	HFR3 65 K	HFR3 69 T	HFR3 93 V	HCDR3 105 L	LFR1 154 G	LFR3 219 E
1	9a										Q		
2a	B15a							Q			Q		
2b	13A4			A				R			V		
	34G1										R		
	15A7							Q			Q		
	21F3	Q*						R	S		G		
	23G5					Q*	E	R			G		
	24D1	Q*						Q			Q		G
	2E1					Q*		Q			G		
	17G1		S						A		Q		
	19F5					Q*		N		M	G		
	20A8							Q					
	10B15							Q			Q		
	12G1										R		
	28C3							Q*			Q		
	29D3	Q*									G		
	31B5	Q*						Q			G		
	32E.5					Q*		Q			G		
	33D6							N			G		
	HV				Y			Q			G	E	

b

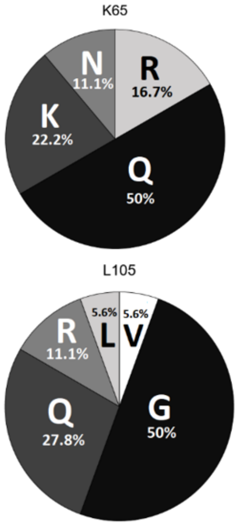

c

```
      1                          HCDR1                    HCDR2    61
RU1   QVNLRESGGGLVQPGGSLRLSCAASGFSFGSYGMHWVRQAPGKGLEWVAVISYGGGNKYYA
10FG2 ---------------------------------------------------------S---
HV    ----------------------------Y----------------------------S---

      62                                      HCDR3           122
RU1   DSVKGRFTISRDNSKNTLYLQMNSLRAEDTAVYYCAKDARDCLLCADWHFDLWGRGTLVTV
10FG2 -------------------------------------------S--T--------------
HV    ---Q-------------------------------------G-S--T--------------

      123                                   LCDR1           183
RU1   SSGGGGSGGGGSGGGGSNFMLTQPHSASGTPGQRVTISCSGSSSNIGSNTVNWYRHLPGSA
10FG2 -------------------------------------------------------------
HV    -------------------------------E-----------------------------

      184  LCDR2                                     LCDR3      244
RU1   PELLIGSHNQRPSGVPDRFSASKSDTSASLAISGLQSEDEADYYCAAWDDSLIGYVFGTGT
10FG2 -------------------G---G---------------------------TL--------
HV    -------------------G---G---------------------------TL--------

      245                         273
RU1   KLTVLGAAAEQKLISEEDLNGAAHHHHHH
10FG2 -----------------------------
HV    -----------------------------
```

Figure S4. Analysis of sequenced clones from the 3rd round of affinity maturation process 2b screening. (a) Changes in the variants generated in the three maturation processes. The empty spaces represent the same amino acid as in 10FG2. Asterisks indicate that a glutamine-encoding amber (TAG) codon was generated. **(b)** Pie chart representing the frequency and percentage of appearance of amino acids at positions K65 and L105 of the 18 scFvs sequenced from maturation process 2b. **(c)** Alignment of the sequences of the scFvs RU1, 10FG2, and HV. At the top of the sequences, the corresponding positions of the different CDRs are indicated. The sequence with thinner letters corresponds to the linker.

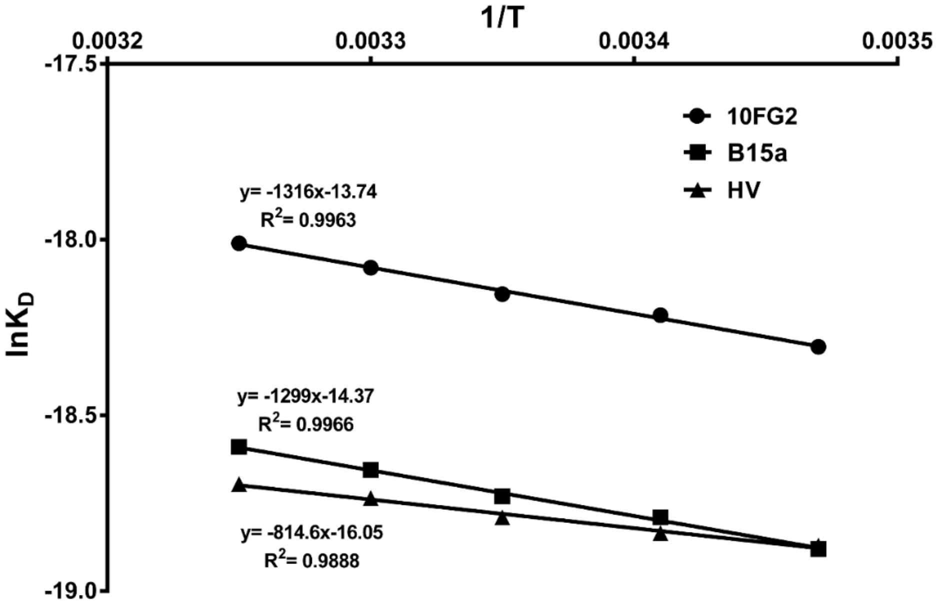

Figure S5. Van't Hoff plot of Chui5-scFvs complexes. The lnK_D of scFvs 10FG2, B15a and HV with Chui5 toxin vs 1/T at different temperatures were plotted. By means of the lines shown and the values of the equation of the straight line obtained by linear regression, the thermodynamic parameters can be calculated. For each straight line, its equation and its multiple correlation coefficient value (R^2) are indicated.
